# The individual and common repertoire of DNA-binding transcriptional regulators of *Corynebacterium glutamicum*, *Corynebacterium efficiens*, *Corynebacterium diphtheriae *and *Corynebacterium jeikeium *deduced from the complete genome sequences

**DOI:** 10.1186/1471-2164-6-86

**Published:** 2005-06-07

**Authors:** Iris Brune, Karina Brinkrolf, Jörn Kalinowski, Alfred Pühler, Andreas Tauch

**Affiliations:** 1 Institut für Genomforschung, Centrum für Biotechnologie, Universität Bielefeld, Universitätsstr. 25, D-33615 Bielefeld, Germany; 2 International NRW Graduate School in Bioinformatics and Genome Research, Centrum für Biotechnologie, Universität Bielefeld, Universitätsstr. 25, D-33615 Bielefeld, Germany; 3 Lehrstuhl für Genetik, Fakultät für Biologie, Universität Bielefeld, Universitätsstr. 25, D-33615 Bielefeld, Germany

## Abstract

**Background:**

The genus *Corynebacterium *includes Gram-positive microorganisms of great biotechnologically importance, such as *Corynebacterium glutamicum *and *Corynebacterium efficiens*, as well as serious human pathogens, such as *Corynebacterium diphtheriae *and *Corynebacterium jeikeium. *Although genome sequences of the respective species have been determined recently, the knowledge about the repertoire of transcriptional regulators and the architecture of global regulatory networks is scarce. Here, we apply a combination of bioinformatic tools and a comparative genomic approach to identify and characterize a set of conserved DNA-binding transcriptional regulators in the four corynebacterial genomes.

**Results:**

A collection of 127 DNA-binding transcriptional regulators was identified in the *C. glutamicum *ATCC 13032 genome, whereas 103 regulators were detected in *C. efficiens *YS-314, 63 in *C. diphtheriae *NCTC 13129 and 55 in *C. jeikeium *K411. According to amino acid sequence similarities and protein structure predictions, the DNA-binding transcriptional regulators were grouped into 25 regulatory protein families. The common set of DNA-binding transcriptional regulators present in the four corynebacterial genomes consists of 28 proteins that are apparently involved in the regulation of cell division and septation, SOS and stress response, carbohydrate metabolism and macroelement and metal homeostasis.

**Conclusion:**

This work describes characteristic features of a set of conserved DNA-binding transcriptional regulators present within the corynebacterial core genome. The knowledge on the physiological function of these proteins should not only contribute to our understanding of the regulation of gene expression but will also provide the basis for comprehensive modeling of transcriptional regulatory networks of these species.

## Background

Upon completion and annotation of the nucleotide sequence of a bacterial genome, a great scientific challenge is to elucidate the regulation of expression of all the predicted genes and to deduce thereof the entirety of regulatory networks present in the respective microorganism. An important prerequisite in understanding regulation of gene expression on the global scale is the identification of the repertoire of regulatory proteins in a genome sequence [1]. DNA-binding transcription factors are key components in the regulation of gene expression because they rapidly respond to changes in the cellular environment by modulating the expression of relevant genes. Three-dimensional structure analyses and amino acid sequence comparisons of DNA-binding transcription factors enabled their allocation into distinct protein classes that apparently use specific structural motifs for DNA recognition and binding [2]. The helix-turn-helix (HTH) motif is obviously the most widely distributed DNA-binding domain in prokaryotic proteins and provides the structural basis for efficient protein-DNA interactions [3]. Although a considerable amino acid sequence divergence has been observed among HTH proteins, they generally share a site-specific DNA-binding domain that is composed of two α-helices separated by a short turn of variable length [4]. Both α-helices are involved in the DNA-binding and recognition process in such a way that the first helix associates non-specifically with the DNA molecule while the second helix recognizes and binds specifically to its cognate operator sequence [5]. Based on variations of the three-dimensional structure of the HTH motif, DNA-binding transcription factors can be subdivided into several DNA-binding domain types [6]. The canonical 'winged helix' type, for instance, consists of two wings, three α-helices and three β-strands and is present in several families of DNA-binding transcription factors. In this case, the third α-helix is the DNA recognition helix, whereas the first and second ones are involved in stabilizing the DNA-binding and recognition process [3]. An interesting outcome of comparative studies between DNA-binding transcription factors was a position-function correlation such that repressor proteins usually possess the HTH motif within the N-terminal region, whereas activators tend to have the HTH motif close to the C-terminal end of the protein [1]. Moreover, the putative physiological role of a DNA-binding transcription factor can be deduced from its classification into an evolutionary regulatory protein family, since within many families the members are homogenous in respect of their regulatory role and the physiology of the regulated genes [1].

Because of the importance of corynebacteria in biotechnology as well as in human medicine they represent an attractive target to elucidate and compare the repertoire of DNA-binding transcription factors by bioinformatic approaches. *Corynebacterium glutamicum *and its closest phylogenetic relative *Corynebacterium efficiens *are both widely known for their capacity to produce amino acids by large-scale fermentation processes [7,8]. On the other hand, *Corynebacterium diphtheriae *is the etiological agent of the acute, communicable disease diphtheria and apparently the most important human pathogen of the genus *Corynebacterium *[9], which also includes a growing number of nosocomial pathogens, such as the multiresistant *Corynebacterium jeikeium *[10]. Accordingly, complete genome sequences of the four corynebacterial species have been determined and annotated recently [11-15]. Subsequent synteny analyses of the predicted coding sequences revealed that the four corynebacteria have largely maintained an ancestral genome structure [15,16]. Therefore, we were not only interested in the identification and classification of the individual DNA-binding transcriptional regulatory repertoire of each of the four corynebacterial species by means of different bioinformatic tools but also in comparative genomic approaches to deduce thereof the common set of regulatory genes. The knowledge on conserved regulatory genes encoded by the corynebacterial core genome certainly provides a solid basis for the future analysis of transcriptional regulatory networks in pathogenic and non-pathogenic corynebacteria.

## Results and discussion

### The repertoire of DNA-binding transcriptional regulators in corynebacterial genomes

To fully understand the regulation of gene expression in a bacterial cell it is necessary to identify and characterize the repertoire of DNA-binding transcriptional regulators with respect to regulatory and physiological properties. Therefore, we have screened the complete genome sequences of four corynebacteria by different bioinformatic tools to detect genes encoding DNA-binding transcriptional regulators. Other gene products typically exhibiting regulatory properties on the transcriptional level, such as sigma factors or two-component systems, were excluded from the present study. The work flow for the identification and analysis of transcriptional regulatory proteins is described in detail in the Methods section. Following three consecutive steps of data collection, a total number of 348 DNA-binding transcriptional regulators were identified in the genomes of *C. glutamicum *ATCC 13032, *C. efficiens *YS-314, *C. diphtheriae *NCTC 13129 and *C. jeikeium *K411. A compilation of the data characterizing the DNA-binding transcriptional regulators of each species is provided as supplementary material (see Additional files 1,2,3 and 4).

An overview of the resulting data along with a genome comparison between the four corynebacterial species is presented in Table 1. A collection of 127 DNA-binding transcriptional regulators was identified in the genome of *C. glutamicum*, whereas 103 transcriptional regulators were identified in the chromosomal sequence of *C. efficiens*, 63 in *C. diphtheriae *and only 55 in *C. jeikeium*. Three additional genes encoding DNA-binding transcriptional regulators were detected on the endogenous plasmid pCE3 of *C. efficiens *YS-314 (data not shown). Considering the differences in genome size of the four corynebacterial species, it became apparent that a larger number of coding regions within a genome sequence requires more genes that encode DNA-binding transcriptional regulators (Table 1). The identified regulatory proteins represent 4.2 and 3.5 %, respectively, of the predicted coding sequences in the genomes of the non-pathogenic corynebacteria *C. glutamicum *and *C. efficiens*, whereas the percentage is reduced to 2.7 and 2.6 %, respectively, in the pathogenic corynebacterial species *C. diphtheriae *and *C. jeikeium*. These data are consistent with a previous observation that an increase of genomic complexity and physiological functionality is generally associated with a more complex regulation of gene expression since the additional genetic information has to be integrated into the existing regulatory networks that operate in a bacterial cell [17].

**Table 1:** Comparison of sequenced corynebacterial genomes

Feature	*C. glutamicum*	*C. efficiens*	*C. diphtheriae*	*C. jeikeium*
	ATCC 13032	YS-314	NCTC 13129	K411
Genome size	3,282,708 bp	3,147,090 bp	2,488,635 bp	2,462,499 bp
Number of coding sequences	3,002	2,950	2,320	2,104
Number of regulators	127	103	63	55
Percentage of regulators	4.2 %	3.5 %	2.7 %	2.6 %

### Classification of corynebacterial DNA-binding transcriptional regulators into regulatory protein families

**Figure 1:**
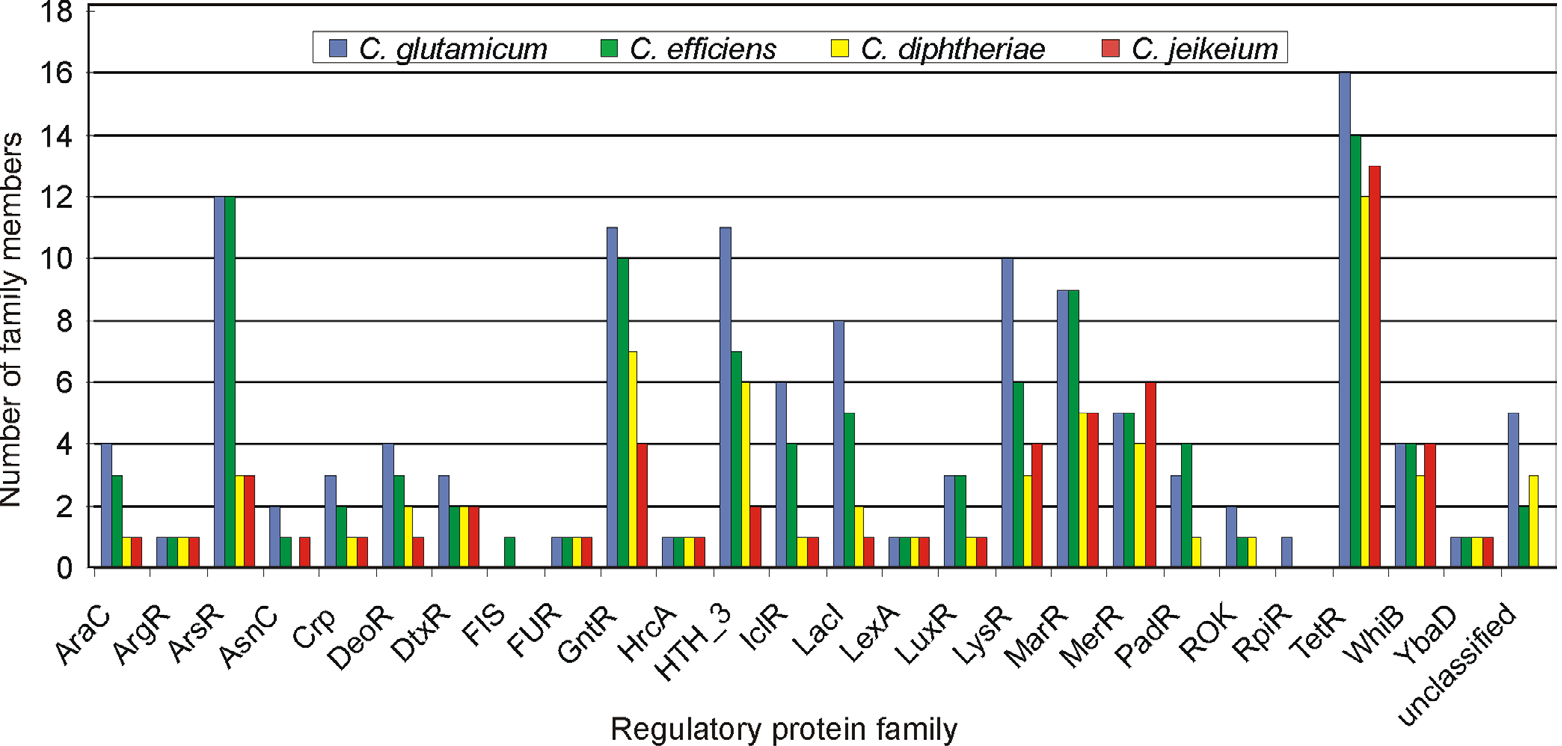
Classification of DNA-binding transcriptional regulators of corynebacteria into regulatory protein families. The identified regulatory protein families are indicated along with the number of assigned family members. The rightmost columns of the diagram comprise a small number of transcriptional regulators that remained unclassified. The regulatory protein families were named according to designations by the Pfam database.

DNA-binding transcriptional regulators can be grouped into evolutionary regulatory protein families based on their amino acid sequence similarity [1]. According to amino acid sequence alignments performed with the CLUSTAL X program, the complete collection of corynebacterial transcriptional regulators fell into 25 protein families with only a small number of regulators that remained unclassified (Figure 1). Most of the identified regulatory protein families can be regarded as homogenous with respect to the size distribution of the assigned family members (see Addional files 1, 2, 3 and 4). At least in some cases the high degree of amino acid sequence similarities along with the conserved protein size within a regulatory protein family indicate that the respective family members derived from a common ancestor. Therefore, one can assume that members of homogenous regulatory protein families tend to affect the expression of genes involved in related physiological functions of the cell [1]. On the other hand for instance, the HTH_3 family contains several members of rather heterogenous size (see Addional files 1, 2, 3 and 4) reflecting a lower degree of similarity of proteins within this family. Accordingly, a more diverse evolutionary history and thus physiological functionality in corynebacteria can be predicted for the members of the HTH_3 family.

The identified regulatory protein families vary significantly in their number of representatives, ranging from TetR, the largest family with up to 16 members, to protein families with a single member, for instance ArgR, FUR, HrcA, LexA and YbaD (Figure 1). The protein families ArgR, HrcA and LexA are characterized in bacterial genomes predominantly by single representatives that are involved in the regulation of arginine metabolism, the heat shock response and the SOS repair pathway of the cell respectively [18]. Most of the regulatory protein families with a few members have homologs in the four corynebacterial genomes with the exception of the PadR and ROK families that are absent in *C. jeikeium *(Figure 1). Further exceptions are single regulators grouped into the RpiR family of *C. glutamicum *and into the FIS family of *C. efficiens*.

In principle, the overall composition of the repertoire of DNA-binding transcriptional regulators identified in the non-pathogenic corynebacteria *C. glutamicum *and *C. efficiens *seemed to be very similar, which was obvious when considering the close phylogenetic relationship of both species [8]. The average number of members per regulatory protein family was calculated as 4.1 for *C. efficiens *and 5.1 in case of *C. glutamicum*, suggesting that species-specific differences are mainly caused by slightly smaller numbers of regulatory proteins per family in *C. efficiens*. On the other hand, the average number of members per regulatory protein family is in the order of 2.6 for the pathogenic corynebacteria *C. diphtheriae *and *C. jeikeium*. Remarkable differences between the transcriptional regulatory repertoire of pathogenic and non-pathogenic corynebacteria became apparent when comparing, for instance, the number of proteins grouped into the ArsR, IclR and LacI families (Figure 1). The ArsR family of transcriptional regulators contains metalloregulatory proteins that control genes whose expression is linked to stress-inducing concentrations of heavy metal ions [19], whereas members of the IclR and LacI families generally respond to environmental changes that affect the carbohydrate metabolism of the cell [20]. It certainly makes sense that soil bacteria have a large diversity of DNA-binding transcriptional regulators that function as metal sensors or respond to changes in the carbohydrate composition of the environment. The larger number of proteins grouped into the IclR and LacI families may provide these bacteria the ability to grow in the presence of several carbon sources and to rapidly adapt their gene expression to changing nutrient conditions. The observed differences in the number of transcriptional regulators involved in carbohydrate metabolism may also reflect the fact that pathogenic bacteria do not require a versatile sugar metabolism since only a limited range of carbohydrate nutrients might be present in their natural habitats. Variations in the number of metalloregulatory sensors may also be linked to the different environmental conditions such that pathogenic bacteria predominantly import metal ions directly from the host rather than from inanimate environment. It is noteworthy that the number of regulatory proteins of the TetR family is otherwise not reduced significantly in the pathogenic species (Figure 1). This observation implies that the TetR repressor family provides a very common switch for the regulation of gene expression in corynebacteria.

### Classification of corynebacterial transcriptional regulators according to DNA-binding domain types

The transcriptional regulators were additionally grouped according to DNA-binding domain types that specify variations of the three dimensional structure of the HTH motif. The DNA-binding domain types were detected by using the domain assignment server SUPERFAMILY [21]. We found that only six DNA-binding domain types have representatives in corynebacteria with the 'winged helix' type being the most prominent HTH motif (Table 2). Especially the hairpin wings of the 'winged helix' display considerable flexibility in their utilization for DNA-binding and alternative modes of DNA recognition [3]. Other DNA-binding domains were also identified in corynebacteria, but only in a few regulatory proteins (Table 2). This includes DNA-binding transcriptional regulators of the WhiB protein family that were suggested to act as transcriptional activators [22]. Structure-function predictions for WhiB proteins indicated that the most C-terminal α-helix is a likely candidate for DNA-binding. Additionally, an aminoterminal Zinc β-ribbon domain combined with an ATP-cone domain was detected in the single member of the YbaD protein family, which is probably involved in a hitherto unknown mechanism of transcriptional regulation of ribonucleotide reductase expression in bacteria [23].

**Table 2:** Domain architecture of corynebacterial DNA-binding transcriptional regulators

DNA-binding domain type	*C. glutamicum*	*C. efficiens*	*C. diphtheriae*	*C. jeikeium*
	ATCC 13032	YS-314	NCTC 13129	K411
Winged helix	73	60	32	26
Homeodomain-like	20	17	14	14
λ repressor-like	19	12	8	3
Putative DNA-binding domain	5	5	4	6
C-terminal effector domain	3	3	1	1
FIS-like	-	1	-	-
C-terminal α-helix*	4	4	3	4
Zinc β-ribbon	1	1	1	1
Unclassified	2	-	-	-

* Putative DNA-binding domain named according to Soliveri *et al*.[22].

Subsequently, the position of the DNA-binding domain within the transcriptional regulators in combination with the regulatory protein family membership was used to predict whether a protein is expected to act as repressor or activator of gene expression. The position of the DNA-binding motif was determined by both the HTH prediction tool [24] and the domain assignment server SUPERFAMILY [21]. This computational approach resulted in an average distribution of DNA-binding transcriptional regulators in the four corynebacterial species of 73.8 % repressors, 16.4 % activators and 9.8 % dual regulators. Dual transcriptional regulators are either activators of several genes and repressors of their own synthesis or activators and repressors of different sets of genes [1]. In particular, the members of the AsnC, Crp and LysR protein families represent dual regulators of gene expression whereas the AraC, LuxR, MerR, and WhiB protein families were regarded as activators of gene expression. However, one has to keep in mind that some regulatory protein families, for instance IclR, MarR and MerR, may include both activator and repressor proteins. Nevertheless, considering that approximately three-quarters of the DNA-binding transcriptional regulators are repressor proteins it is apparent that mechanistic requirements for repression are dominant in the architecture of regulatory networks in corynebacteria. A similar analysis of 314 regulatory proteins of *Escherichia coli *revealed a different picture in such a way that a quite even distribution of 42.8%, 34.8% and 22.3% of repressors, activators and dual regulators was predicted for this species [1].

### The common repertoire of DNA-binding transcriptional regulators identified in four corynebacterial genome sequences

**Figure 2:**
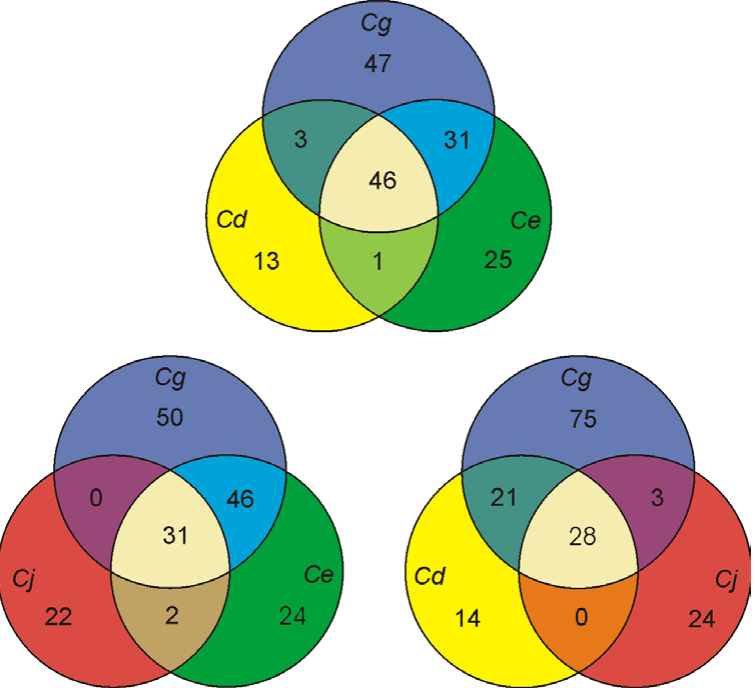
Comparative content analysis of genes encoding DNA-binding transcriptional regulators in sequenced corynebacterial genomes. The Venn diagrams show the number of shared and species-specific genes among the four genomes. Abbreviations: *Cg*, *C. glutamicum *ATCC 13032; *Ce*, *C. efficiens *YS-314; *Cd*, *C. diphtheriae *NCTC 13129; *Cj*, *C. jeikeium *K411.

We were then interested to specify those DNA-binding transcriptional regulators that are common in the four sequenced corynebacterial genomes. For this purpose, orthologous proteins were identified in the complete collection of DNA-binding transcriptional regulators by using the BLASTP algorithm to detect amino acid sequence similarities and by performing synteny analyses of the respective genomic context. This comparative content analysis of transcriptional regulators allowed us to calculate the number of shared and species-specific regulatory proteins among the four corynebacteria. The resulting data were summarized in the Venn diagrams of Figure 2. It is striking that the genomes of *C. glutamicum *and *C. efficiens *share 77 transcriptional regulators suggesting that many regulatory networks might be conserved in both species and controlled by homologous regulatory functions. This is consistent with the very similar distribution of DNA-binding transcriptional regulators of both species within the identified regulatory protein families (Figure 1). Approximately 75 % of the transcriptional regulators detected in *C. diphtheriae *are shared with the repertoire of regulatory proteins of the non-pathogenic corynebacteria *C. glutamicum *and *C. efficiens *(Figure 2). Consequently, both species provide valuable model systems for investigating the regulation of gene expression in *C. diphtheriae *since it is likely that not only the regulatory proteins but also the corresponding regulatory networks are somehow conserved. The composition of the small regulatory repertoire of *C. jeikeium *is much more intriguing since virtually all of the DNA-binding transcriptional regulators are either species-specific or shared with the other three corynebacteria. This finding might reflect the distant phylogenetic relationship between *C. jeikeium *and the investigated corynebacterial species [25]. By combining the data of the comparative content analyses, the common set of DNA-binding transcriptional regulators present in *C. glutamicum*, *C. efficiens*, *C. diphtheriae *and *C. jeikeium *was finally confined to only 28 proteins (Table 3).

**Table 3:** The common set of DNA-binding transcriptional regulators in corynebacteria

Functional category	CDS in *C. glutamicum *ATCC 13032		Orthologous CDS in		
	
	No.	Gene name or regulator family	*C. efficiens *YS-314	*C. diphtheriae *NCTC 13129	*C. jeikeium *K411
Cell division & septation	*cg0878*	*whiB1*	*ce0783*	*dip0712*	*jk1618*
	*cg0850*	*whiB2*	*ce0758*	*dip0684*	*jk1644*
	*cg0337*	*whiB4*	*ce0283*	*dip0299*	*jk1976*

SOS & stress response	*cg2109*	*oxyR*	*ce1817*	*dip1421*	*jk1102*
	*cg2114*	*lexA*	*ce1823*	*dip1426*	*jk1106*
	*cg2152*	*clgR*	*ce1855*	*dip1456*	*jk1122*
	*cg2516*	*hrcA*	*ce2190*	*dip1721*	*jk0600*
	*cg3097*	*hspR*	*ce2626*	*dip2117*	*jk0184*
	*cg1765*	ArsR family	*ce1687*	*dip1296*	*jk0985*

Macroelement & metal	*cg2103*	*dtxR*	*ce1812*	*dip1414*	*jk1097*
homeostasis	*cg2502*	*furB*	*ce2180*	*dip1710*	*jk0612*
	*cg3253*	*mcbR*	*ce2788*	*dip2274*	*jk0101*
	*cg1631*	MerR family	*ce1574*	*dip1205*	*jk0904*
	*cg1633*	MerR family	*ce1576*	*dip1207*	*jk0906*

Carbohydrate metabolism	*cg0350*	*glxR*	*ce0287*	*dip0303*	*jk1972*
	*cg0444*	*ramB*	*ce0385*	*dip0369*	*jk1934*
	*cg1738*	*acnR*	*ce1663*	*dip1284*	*jk0970*
	*cg2115*	DeoR family	*ce1824*	*dip1427*	*jk1107*
	*cg1486*	IclR family	*ce1426*	*dip1126*	*jk1222*
	*cg2910*	LacI family	*ce2511*	*dip1969*	*jk0329*

Biosynthesis pathways	*cg1585*	*argR*	*ce1531*	*dip1172*	*jk0846*
	*cg2112*	YbaD family	*ce1820*	*dip1424*	*jk1105*

Unknown	*cg3261*	GntR family	*ce2809*	*dip2280*	*jk0088*
	*cg2831*	LuxR family	*ce2445*	*dip1889*	*jk0397*
	*cg3001*	MarR family	*ce2556*	*dip2008*	*jk0271*
	*cg3315*	MarR family	*ce2826*	*dip2296*	*jk2061*
	*cg0454*	TetR family	*ce0397*	*dip0937*	*jk1455*
	*cg1053*	TetR family	*ce0985*	*dip0888*	*jk1501*

According to functional assignments deduced from computational predictions and protein similarities, 22 of the common DNA-binding transcriptional regulators were grouped into five functional categories (Table 3). The physiological role of the remaining six transcriptional regulators remained unknown since the potential targets of their regulation were difficult to predict from the existing data and the genome organization. The resulting functional classification is of particular interest since members of four categories are apparently involved in the control of fundamental processes of the bacterial life style, namely cell division and septation, SOS and stress response, carbohydrate metabolism and macroelement and metal homeostasis (Table 3). A fifth category comprises only single representatives of the ArgR family and the YbaD family that are involved in the regulation of specific biosynthesis pathways [18,23].

#### Cell division and septation

The first functional category of common DNA-binding transcriptional regulators includes three members of the WhiB protein family. This regulatory protein family represents a specific group of transcriptional activators that appeared to be present in perhaps all actinobacteria while being absent from all other sequenced bacterial genomes [22]. Members of the WhiB protein family were postulated to function in cell division and septation of mycobacteria [26] and in differentiation of streptomycetes [27], possibly by sensing redox changes in the environment or internally during metabolic shifts that occur as inescapable part of alterations in the cellular metabolism. The genomes of *C. glutamicum*, *C. efficiens *and *C. jeikeium *carry four *whiB *homologs while a *whiB3 *ortholog is missing in the *C. diphtheriae *genome. In this context it is noteworthy that the *whiB3 *gene was shown to be dispensable for growth of *Mycobacterium tuberculosis *and *Mycobacterium smegmatis *[28,29].

#### SOS and stress response

The second functional category contains six DNA-binding transcriptional regulators that are apparently involved in the SOS and stress response of the cell. First of all, homologs of the LexA protein and the redox-responsive transcriptional regulator OxyR were identified in the four corynebacterial genomes. These proteins are well-known to control the SOS response and the oxidative stress response of the bacterial cell respectively [30,31]. Moreover, the conserved HrcA, HspR and ClgR proteins were included in this functional category since they represent main components of the heat shock response of *C. glutamicum *[32,33]. In general, the major function of the three responses is either the repair or the elimination of damaged macromolecules of the cell. A conserved member of the ArsR protein family was also included into this functional category of transcriptional regulators because its conserved genomic context suggested that it plays a role in the regulation of expression of the corynebacterial *suf *gene cluster. The respective genes of *Escherichia coli *have been implicated in the assembly of Fe-S clusters in proteins during oxidative stress conditions by encoding a specific sulphur transfer mechanism that limits the release of sulphide and thus the formation of highly damaging hydroxyl radicals [34].

#### Macroelement and metal homeostasis

The third category of conserved DNA-binding transcriptional regulators is obviously involved in the regulation of macroelement and metal homeostasis of corynebacteria. This functional category of proteins is build up by the McbR, DtxR and FurB homologs and includes also two regulators of the MerR family which is a collection of metal-responsive transcriptional activators [35]. The McbR protein of *C. glutamicum *was recently demonstrated to regulate a wide spectrum of genes comprising all aspects of transport and metabolism of the macroelement sulphur in this species as well as two transcriptional regulators that were classified into the ROK family [36,37]. In this context it is noteworthy that AmtR, the master regulator of nitrogen metabolism of *C. glutamicum *[38], was not among the conserved set of DNA-binding transcriptional regulators since an orthologous gene is apparently absent in the genome of *C. jeikeium*. This striking difference in the equipment of corynebacterial genomes with global regulatory genes indicated that transcriptional regulation of sulphur metabolism is vitally important in corynebacteria in contrast to a conservation of transcriptional control mechanisms to provide an optimal supply of nitrogen for cellular processes. The DtxR repressor is known as global regulator of iron homeostasis in *C. diphtheriae *[39] but its regulatory network might also include genes whose expression is linked to protect the cell from oxidative stress [40]. The conserved FurB proteins were included in this functional category due to the observation that expression of the orthologous gene in *Mycobacterium tuberculosis *was specifically induced by zinc [41].

#### Carbohydrate metabolism

Our data suggested that six conserved genes are involved in the regulation of corynebacterial carbohydrate metabolism. The respective functional category of DNA-binding transcriptional regulators includes the AcnR repressor that controls the expression of the aconitase gene *acn*, thus representing an important control point of the tricarboxylic acid cycle of *C. glutamicum *[42]. Both RamB and GlxR apparently participate in the regulation of acetate metabolism and the glyoxylate bypass of *C. glutamicum *[43,44]. The GlxR protein represses at least the expression of the *aceB *gene in the presence of cAMP, whereas RamB seems to be a more global regulator of gene expression. The observation that the *glxR *gene could not be mutated in *C. glutamicum *implies that the common set of DNA-binding transcriptional regulators may include further members with essential regulatory functions. Three transcriptional regulators of the DeoR, IclR and LacI families were also grouped into this functional category due to the conserved physiological role of these homogenous regulatory protein families in the control of carbohydrate metabolism [20,45,46].

## Conclusion

Based on genome analyses with different bioinformatic tools we have defined the individual set of DNA-binding transcriptional regulators in four sequenced corynebacterial genomes and deduced thereof the common repertoire of transcriptional regulators of these species. The data will provide valuable information on the corynebacterial biology in general and the ways these bacteria interact with different environments by modulating the expression of relevant genes. Only 28 DNA-binding transcriptional regulators have counterparts in the four corynebacteria and according to functional predictions most of these proteins are involved in the control of fundamental cellular processes encoded by the corynebacterial core genome. However, the majority of the genes belonging to the common set of transcriptional regulators has not been adequately studied yet, so further research and direct functional studies are necessary to determine the physiological role of these regulators and to precisely place them into the architecture of corynebacterial transcriptional regulatory networks. Further research is also necessary to determine the physiological function of species-specific and shared transcriptional regulators that might be involved either in the regulation of cellular processes relevant for biotechnological production or that might control the expression of genes involved for instance in virulence of pathogenic corynebacteria. With respect to a very recent study on the regulation of sulphur metabolism by the conserved transcriptional regulator McbR [37] we are confident that the combined use of classical genetic approaches along with post-genomic techniques, such as DNA microarrays and 2D gel electrophoresis, will provide the frame to decipher transcriptional regulatory networks and to build comprehensive regulatory models of corynebacterial cells.

## Methods

**Figure 3:**
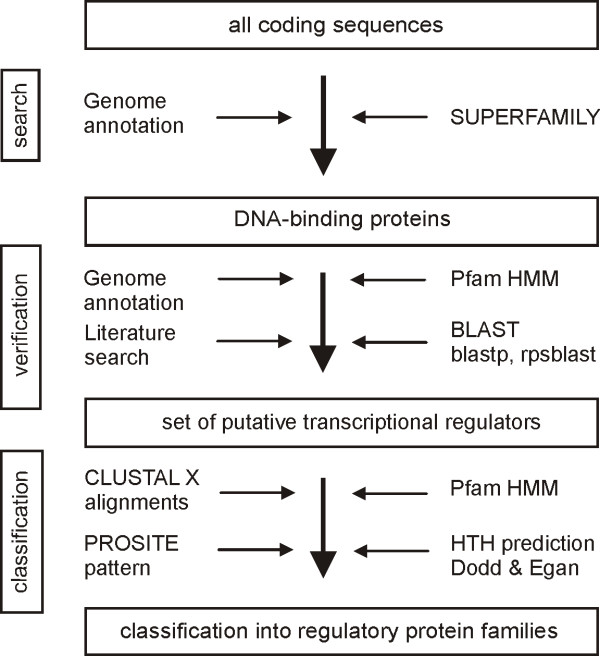
Work flow applied for the identification and classification of DNA-binding transcriptional regulators in corynebacterial genomes. The approach includes several methods and tools and consists of three consecutive steps indicated on the left.

The general strategy to detect and classify DNA-binding transcriptional regulators of sequenced corynebacterial genomes was based on a combination of several bioinformatic tools. According to the work flow schematized in Figure 3, putative DNA-binding proteins were first searched for in the complete genome sequences of four corynebacterial species, comprising *C. glutamicum *ATCC 13032 (GenBank accession number BX927147), *C. efficiens *YS-314 (BA000035), *C. diphtheriae *NCTC 13129 (BX248353) and *C. jeikeium *K411 (CR931997). These searches were performed by means of the genome assignment server SUPERFAMILY [21] that contains a library of hidden Markov models (HMMs) and the results of searches by these models against completely sequenced genomes. The HMMs of SUPERFAMILY are based on the sequences of domains collected in the Structural Classification of Proteins (SCOP) database [47] and are thus applicable for a structural classification of proteins. The search for corynebacterial DNA-binding proteins also included information deduced from the respective genome annotations deposited in public databases [12-14]. To identify among the DNA-binding proteins those potentially representing transcriptional regulators, 35 different HMM profiles of bacterial protein families with known function in transcriptional regulation of gene expression were downloaded from the Pfam database and used for searches against the predicted corynebacterial proteins by applying the HMMsearch module of the HMMER software package [48]. Verification of the results was performed by means of the BLAST algorithms [49], in particular by blastp and rpsblast along with the NCBI Conserved Domain Search program. Literature information was used to find additional coding sequences for DNA-binding transcriptional regulators in the corynebacterial genome sequences. During the final step of data analysis, the putative DNA-binding transcriptional regulators were grouped into regulatory protein families. This classification was performed by using HMM profiles of the Pfam database, CLUSTAL X alignments [50] of those proteins belonging to a distinct regulatory family and results of PROSITE pattern searches [45]. The HTH recognition tool designed by Dodd and Egan [24] was used to scan the putative DNA-binding transcriptional regulators for the presence and position of HTH motifs. By combining all these data, defined collections of putative DNA-binding transcriptional regulators were identified for each of the selected corynebacterial genomes (see Additional files 1,2,3 and 4). To deduce thereof the common set of DNA-binding transcriptional regulators of the four sequenced corynebacterial species, comparative genomic analyses were performed. This approach included blastp searches with the identified regulatory proteins [49] against the predicted proteins of each genome sequence, using an evalue cut off smaller than 1 × e^-10^. To verify whether the orthologous regulatory genes are present in the same genomic context, neighbouring coding sequences were included into synteny analyses.

## Authors' contributions

IB carried out the bioinformatic analyses, comparative genomics studies and drafted the manuscript. KB performed all analyses related to the *C. diphtheriae *genome sequence. JK participated in data evaluation and helped to draft the manuscript. AP participated in coordination and supervison and conceived of the design of the figures. AT conceived of the study and participated in its coordination. All authors read and approved the final manuscript.

## Supplementary Material

Additional File 1 classification and relevant molecular data of the DNA-binding transcriptional regulators identified in *C. glutamicum *ATCC 13032. Click here for file

Additional File 4 classification and relevant molecular data of the DNA-binding transcriptional regulators identified in *C. jeikeium *K411. Click here for file

Additional File 2 classification and relevant molecular data of the DNA-binding transcriptional regulators identified in *C. efficiens *YS-314. Click here for file

Additional File 3 classification and relevant molecular data of the DNA-binding transcriptional regulators identified in *C. diphtheriae *NCTC 13129. Click here for file
