# Supplementary material for: The individual and common repertoire of DNA-binding transcriptional regulators of Corynebacterium glutamicum, Corynebacterium efficiens, Corynebacterium diphtheriae and Corynebacterium jeikeium deduced from the complete genome sequences
Source: BMC Genomics. 2005 Jun 7;6:86. doi: 10.1186/1471-2164-6-86 (PMC1180825; doi:10.1186/1471-2164-6-86)
Supplement: Additional File 1 — classification and relevant molecular data of the DNA-binding transcriptional regulators identified in C. glutamicum ATCC 13032. [file 1471-2164-6-86-S1.pdf]

Additional file 1

**DNA-binding transcriptional regulators identified in *C. glutamicum* ATCC 13032**								
			Gene		Protein		DNA-binding domain	
No.	Regulator family	Number of family members	No.	Name	Length [amino acids]	Molecular mass [kDa]	Type	Position
1	AraC	4	*cg0051*		332	36.1	homeodomain-like*	N, C-terminal
			cg1120		331	36.0	homeodomain-like*	central
			cg2712#		162	17.8	homeodomain-like	central
			cg2965		337	70.9	homeodomain-like	C-terminal
2	ArgR	1	*cg1585*	*argR*	171	18.4	winged helix	N-terminal
3	ArsR	12	*cg0317*		118	12.6	winged helix	central
			cg0527		119	12.9	winged helix	central
			cg0993		104	11.8	winged helix	central
			cg1032		119	12.6	winged helix	central
			cg1211		158	18.1	winged helix	central
			cg1765		228	25.0	winged helix	N-terminal
			cg1831		101	11.1	winged helix	central
			cg2320		592	16.3	winged helix	N-terminal
			cg2500		123	13.3	winged helix	central
			cg2648		165	18.2	winged helix	N-terminal
			cg3082		113	14.4	winged helix	central
			cg3373		112	12.4	winged helix	central
4	AsnC	2	*cg0313*	*lrp*	151	16.7	winged helix	N-terminal
			cg2942		166	18.5	winged helix	N-terminal
5	Crp	3	*cg0350*	*glxR*	227	24.9	winged helix	C-terminal
			cg1327		250	27.4	winged helix	C-terminal
			cg3291		157	17.0	winged helix	central
6	DeoR	4	*cg0139*		260	27.9	winged helix	N-terminal
			cg0146		242	30.8	winged helix	N-terminal
			cg2115		259	27.5	winged helix	N-terminal
			cg2118		264	35.1	winged helix	N-terminal
7	DtxR	3	*cg0741*		223	24.5	winged helix	N-terminal
			cg2103	*dtxR*	228	26.6	winged helix	N-terminal
			cg2784		220	23.7	winged helix	N-terminal
8	FUR	1	*cg2502*	*furB*	144	15.7	winged helix	central
9	GntR	11	*cg0196*		253	32.3	winged helix	N-terminal
			cg0764		240	26.7	winged helix	N-terminal
			cg0897	*pdxR*	453	48.8	winged helix	N-terminal
			cg1143		217	24.0	winged helix	N-terminal
			cg1935		246	26.8	winged helix	N-terminal
			cg2544		240	31.8	winged helix	N-terminal
			cg2783		250	27.2	winged helix	N-terminal
			cg2936		240	27.7	winged helix	N-terminal
			cg3202		266	32.5	winged helix	N-terminal
			cg3224		231	25.0	winged helix	N-terminal
			cg3261		121	13.2	winged helix	N-terminal
10	HrcA	1	*cg2516*	*hrcA*	342	42.3	winged helix	central
11	HTH_3	11	*cg0444*	*ramB*	474	36.2	λ repressor-like	N-terminal
			cg0579		260	11.0	λ repressor-like	N-terminal
			cg0787		124	14.3	λ repressor-like	N-terminal
			cg0800		441	48.9	λ repressor-like	N-terminal
			cg1392		75	8.3	λ repressor-like	central
			cg1464		71	7.7	λ repressor-like	central
			cg1527		325	35.6	λ repressor-like	N-terminal
			cg2040		127	14.1	λ repressor-like	central
			cg2152	*clgR*	107	11.7	λ repressor-like	central
			cg3087		189	20.4	λ repressor-like	N-terminal

			cg3230		127	13.9	λ repressor-like	central
12	IclR	6	*cg0646*		268	29.0	winged helix	N-terminal
			cg1486		235	24.7	winged helix	N-terminal
			cg2624		255	27.6	winged helix	N-terminal
			cg2922		218	23.7	winged helix	N-terminal
			cg3352		258	28.0	winged helix	N-terminal
			cg3388		494	53.1	winged helix*	N, C-terminal
13	LacI	8	*cg0210*		331	35.4	λ repressor-like	N-terminal
			cg0221		330	35.5	λ repressor-like	N-terminal
			cg1410		369	39.3	λ repressor-like	N-terminal
			cg1547		346	36.5	λ repressor-like	N-terminal
			cg2242		327	34.6	λ repressor-like	N-terminal
			cg2314		332	35.4	λ repressor-like	N-terminal
			cg2910		360	33.2	λ repressor-like	N-terminal
			cg2729#		151	16.3	λ repressor-like	N-terminal
14	LexA	1	*cg2114*	*lexA*	253	28.2	winged helix	C-terminal
15	LuxR	3	*cg2627*		687	75.5	C-terminal effector domain	C-terminal
			cg2641		895	99.8	C-terminal effector domain	C-terminal
			cg2831		281	30.8	C-terminal effector domain	C-terminal
16	LysR	10	*cg0019*		298	32.2	winged helix	N-terminal
			cg0272		254	27.2	winged helix	N-terminal
			cg0500		294	32.7	winged helix	N-terminal
			cg0537		294	32.2	winged helix	N-terminal
			cg0702		303	33.5	winged helix	N-terminal
			cg1425	*lysG*	290	31.3	winged helix	N-terminal
			cg2109	*oxyR*	327	35.0	winged helix	N-terminal
			cg2268		286	30.9	winged helix	N-terminal
			cg2899		235	25.9	winged helix	N-terminal
			cg3239		310	33.3	winged helix	N-terminal
17	MarR	9	*cg0112*	*ureR*	171	19.1	winged helix	central
			cg0217		102	11.2	winged helix	central
			cg0343		155	19.3	winged helix	central
			cg0725		195	21.7	winged helix	N-terminal
			cg1324		162	18.6	winged helix	central
			cg2766		164	18.6	winged helix	central
			cg3001		158	16.8	winged helix	central
			cg3246		152	16.8	winged helix	central
			cg3315		157	17.9	winged helix	central
18	MerR	5	*cg1631*		252	27.1	putative DNA-binding domain	central
			cg1633		191	21.2	putative DNA-binding domain	central
			cg2357		334	36.1	putative DNA-binding domain	N-terminal
			cg2889		251	28.8	putative DNA-binding domain	N-terminal
			cg3097	*hspR*	146	16.5	putative DNA-binding domain	N-terminal
19	PadR	3	*cg0979*		169	19.1	winged helix	central
			cg2615		192	21.5	winged helix	N-terminal
			cg3303		192	21.5	winged helix	central
20	ROK	2	*cg0012*		357	38.7	winged helix	N-terminal
			cg0156		381	41.0	winged helix	N-terminal
21	RpiR	1	*cg1648*		293	30.4	winged helix	N-terminal
22	TetR	16	*cg0454*		217	25.8	homeodomain-like	N-terminal
			cg0986	*amtR*	222	24.3	homeodomain-like	N-terminal
			cg1053		153	21.4	homeodomain-like	N-terminal
			cg1098		217	24.0	homeodomain-like	N-terminal
			cg1308		229	25.0	homeodomain-like	N-terminal
			cg1467		207	21.0	homeodomain-like	central
			cg1738	*acnR*	188	21.1	homeodomain-like	N-terminal
			cg1846		198	21.7	homeodomain-like	N-terminal

			cg2309		191	20.9	homeodomain-like	N-terminal
			cg2462		170	14.2	homeodomain-like	central
			cg2614		241	28.3	homeodomain-like	N-terminal
			cg2686		246	27.6	homeodomain-like	N-terminal
			cg2737		203	25.0	homeodomain-like	N-terminal
			cg2894		177	20.2	homeodomain-like	N-terminal
			cg3253	*mcbR*	213	26.3	homeodomain-like	N-terminal
			cg3384		185	21.1	homeodomain-like	N-terminal
23	WhiB	4	*cg0337*	*whiB4*	351	12.8	C-terminal α-helix	C-terminal
			cg0695	*whiB3*	300	11.1	C-terminal α-helix	C-terminal
			cg0850	*whiB2*	315	11.7	C-terminal α-helix	C-terminal
			cg0878	*whiB1*	261	9.5	C-terminal α-helix	C-terminal
24	YbaD	1	*cg2112*		150	18.7	Zinc β-ribbon	N-terminal
25	unclassified	5	*cg0027*		123	13.8	winged helix	N-terminal
			cg0565	*pkwR*	504	55.7	unclassified	C-terminal
			cg1340		230	25.1	winged helix	N-terminal
			cg1552		127	14.1	winged helix	central
			cg2746		394	43.3	unclassified	C-terminal

* Two DNA-binding domains were identified.

# Defective coding sequence.
