# Supplementary material for: The individual and common repertoire of DNA-binding transcriptional regulators of Corynebacterium glutamicum, Corynebacterium efficiens, Corynebacterium diphtheriae and Corynebacterium jeikeium deduced from the complete genome sequences
Source: BMC Genomics. 2005 Jun 7;6:86. doi: 10.1186/1471-2164-6-86 (PMC1180825; doi:10.1186/1471-2164-6-86)
Supplement: Additional File 4 — classification and relevant molecular data of the DNA-binding transcriptional regulators identified in C. jeikeium K411. [file 1471-2164-6-86-S4.pdf]

Additional file 4

**DNA-bindng transcriptional regulators identified in *C. jeikeium* K411**								
			Gene		Protein		DNA-binding domain	
No.	Regulator family	Number of family members	No.	Name	Length [amino acids]	Molecular mass [kDa]	Type	Position
1	AraC	1	*jk0817*		227	27.5	homeodomain-like	C-terminal
2	ArgR	1	*jk0846*	*argR*	150	16.7	winged helix	N-terminal
3	ArsR	3	*jk0985*		227	24.2	winged helix	N-terminal
			jk1437		119	12.7	winged helix	central
			jk2006	*arsR*	115	12.5	winged helix	central
4	AsnC	1	*jk0974*		72	81.7	winged helix	N-terminal
5	Crp	1	*jk1972*	*glxR*	227	25.1	winged helix	N-terminal
6	DeoR	1	*jk1107*		270	28.9	winged helix	N-terminal
7	DtxR	2	*jk1097*	*dtxR*	240	26.9	winged helix	N-terminal
			jk1485	*mntR*	238	26.0	winged helix	N-terminal
8	FUR	1	*jk0612*	*furB*	143	15.7	winged helix	C-terminal
9	GntR	4	*jk0088*		124	13.5	winged helix	C-terminal
			jk0404		214	23.5	winged helix	N-terminal
			jk1156		123	13.0	winged helix	N-terminal
			jk1838		252	28.3	winged helix	N-terminal
10	HrcA	1	*jk0600*	*hrcA*	341	37.2	winged helix	N-terminal
11	HTH_3	2	*jk1122*	*clgR*	118	12.5	λ repressor-like	central
			jk1934	*ramB*	501	56.0	λ repressor-like	N-terminal
12	IclR	1	*jk1222*		244	25.9	winged helix	N-terminal
13	LacI	1	*jk0329*		369	38.2	λ repressor-like	N-terminal
14	LexA	1	*jk1106*	*lexA*	267	28.9	winged helix	N-terminal
15	LuxR	1	*jk0397*		285	31.1	C-terminal effector domain	C-terminal
16	LysR	4	*jk0144*		302	33.3	winged helix	N-terminal
			jk0410		286	30.6	winged helix	N-terminal
			jk1102	*oxyR*	320	33.9	winged helix	N-terminal
			jk1740		304	32.2	winged helix	N-terminal
17	MarR	5	*jk0257*		180	20.1	winged helix	central
			jk0271		148	16.4	winged helix	central
			jk2061		163	18.2	winged helix	central
			jk2072		168	19.0	winged helix	C-terminal
			jk1950		174	19.3	winged helix	N-terminal
18	MerR	6	*jk0184*	*hspR*	138	15.6	putative DNA-binding domain	N-terminal
			jk0904		245	26.6	putative DNA-binding domain	central
			jk0906		170	18.6	putative DNA-binding domain	N-terminal
			jk1418		258	29.6	putative DNA-binding domain	N-terminal
			jk1441		129	14.3	putative DNA-binding domain	N-terminal
			jk1578		134	15.1	putative DNA-binding domain	N-terminal
19	TetR	13	*jk0018*		191	20.5	homeodomain-like	N-terminal
			jk0029		227	25.0	homeodomain-like	N-terminal
			jk0101	*mcbR*	251	27.8	homeodomain-like	N-terminal
			jk0232		239	26.6	homeodomain-like	N-terminal
			jk0323		188	21.4	homeodomain-like	N-terminal
			jk0624		191	20.5	homeodomain-like	N-terminal
			jk0810		203	22.7	homeodomain-like	N-terminal
			jk0970	*acnR*	189	21.2	homeodomain-like	N-terminal
			jk1423		188	20.3	homeodomain-like	N-terminal
			jk1455		232	26.2	homeodomain-like	N-terminal
			jk1500		222	24.3	homeodomain-like	N-terminal
			jk1501		253	27.8	homeodomain-like	N-terminal
			jk1552		204	22.4	homeodomain-like	N-terminal
20	WhiB	4	*jk1618*	*whiB1*	88	9.9	C-terminal α-helix	C-terminal
			jk1644	*whiB2*	121	13.7	C-terminal α-helix	C-terminal

			jk1727	*whiB3*	101	11.2	C-terminal α-helix	C-terminal
			jk1976	*whiB4*	124	13.7	C-terminal α-helix	C-terminal
21	YbaD	1	*jk1105*	*ybaD*	163	18.2	Zinc β-ribbon	N-terminal
