# Supplementary material for: The individual and common repertoire of DNA-binding transcriptional regulators of Corynebacterium glutamicum, Corynebacterium efficiens, Corynebacterium diphtheriae and Corynebacterium jeikeium deduced from the complete genome sequences
Source: BMC Genomics. 2005 Jun 7;6:86. doi: 10.1186/1471-2164-6-86 (PMC1180825; doi:10.1186/1471-2164-6-86)
Supplement: Additional File 2 — classification and relevant molecular data of the DNA-binding transcriptional regulators identified in C. efficiens YS-314. [file 1471-2164-6-86-S2.pdf]

Additional file 2

DNA-binding transcriptional regulators identified in *C. efficiens* YS-314								
			Gene		Protein		DNA-binding domain	
No.	Regulator family	Number of family members	No.	Name	Length [amino acids]	Molecular mass [kDa]	Type	Position
1	AraC	3	*ce0519*		321	34.6	homeodomain-like*	C-terminal
			ce1047		426	46.0	homeodomain-like*	central
			ce2218		335	37.4	homeodomain-like	C-terminal
2	ArgR	1	*ce1531*		190	20.3	winged helix	N-terminal
3	ArsR	12	*ce0009*		142	15.4	winged helix	central
			ce0466		124	13.4	winged helix	central
			ce0874		147	16.0	winged helix	central
			ce0945		133	14.6	winged helix	central
			ce1454		218	23.6	winged helix	central
			ce1534		128	14.0	winged helix	central
			ce1632		176	19.8	winged helix	central
			ce1676		113	12.4	winged helix	central
			ce1687		238	25,7	winged helix	N-terminal
			ce2014		192	21.3	winged helix	C-terminal
			ce2179		136	14.7	winged helix	central
			ce2678		125	13.6	winged helix	central
4	AsnC	1	*ce2745*		336	37.1	winged helix*	N-terminal, central
5	Crp	2	*ce0287*		227	25.0	winged helix	C-terminal
			ce1525		246	26.7	winged helix	C-terminal
6	DeoR	3	*ce1824*		246	26.7	winged helix	N-terminal
			ce1827		301	31.2	winged helix	N-terminal
			ce2836		267	28.8	winged helix	N-terminal
7	DtxR	2	*ce0651*	*sirR*	248	27.5	winged helix	N-terminal
			ce1812	*dtxR*	241	27.1	winged helix	N-terminal
8	FIS	1	*ce0118*		392	43.1	FIS-like	C-terminal
9	FUR	1	*ce2180*	*fur*	175	19.1	winged helix	central
10	GntR	10	*ce1068*		253	28.4	winged helix	N-terminal
			ce1072		254	28.4	winged helix	N-terminal
			ce1130		257	28.5	winged helix	N-terminal
			ce2223		272	30.4	winged helix	N-terminal
			ce2321		263	28.6	winged helix	N-terminal
			ce2422		263	28.6	winged helix	N-terminal
			ce2519		223	25.1	winged helix	N-terminal
			ce2725		265	29.3	winged helix	N-terminal
			ce2757		232	25.3	winged helix	N-terminal
			ce2809		124	13.7	winged helix	C-terminal
11	HrcA	1	*ce2190*		444	47.8	winged helix	central
12	HTH_3	7	*ce0385*		474	53.9	λ repressor-like	N-terminal
			ce0512		260	29.5	λ repressor-like	N-terminal
			ce0705		169	18.9	λ repressor-like	central
			ce1210		378	42.0	λ repressor-like	N-terminal
			ce1390		67	7.5	λ repressor-like	central
			ce1855		108	11.4	λ repressor-like	central
			ce2748		102	10.8	λ repressor-like	central
13	IclR	4	*ce0563*		257	28.1	winged helix	N-terminal
			ce1426		235	24.6	winged helix	N-terminal
			ce2294		269	29.0	winged helix	N-terminal
			ce2861		260	28.5	winged helix	N-terminal
14	LacI	5	*ce0018*		403	43.3	λ repressor-like	N-terminal
			ce1349		403	43.3	λ repressor-like	N-terminal
			ce1485		340	35.8	λ repressor-like	N-terminal
			ce2376		239	41.4	λ repressor-like	N-terminal

			ce2511		359	38.6	λ repressor-like	N-terminal
15	LexA	1	*ce1823*		269	29.3	winged helix	C-terminal
16	LuxR	3	*ce2297*		731	79.5	C-terminal effector domain	C-terminal
			ce2309		895	99.3	C-terminal effector domain	C-terminal
			ce2445		281	30.7	C-terminal effector domain	C-terminal
17	LysR	6	*ce0061*		309	33.7	winged helix	N-terminal
			ce0439		171	17.8	winged helix	N-terminal
			ce0726		312	33.7	winged helix	N-terminal
			ce1358		290	31.5	winged helix	N-terminal
			ce1817		410	44.8	winged helix	central
			ce2767		263	28.6	winged helix	N-terminal
18	MarR	9	*ce0027*		178	19.2	winged helix	N-terminal
			ce0095		195	22.0	winged helix	central
			ce0303		107	11.6	winged helix	central
			ce0644		209	22.7	winged helix	central
			ce1273		162	18.2	winged helix	central
			ce2405		173	19.6	winged helix	central
			ce2556		148	16.8	winged helix	central
			ce2826		153	17.4	winged helix	central
			ce2909		146	16.5	winged helix	central
19	MerR	5	*ce1574*		260	28.0	putative DNA-binding domain	central
			ce1576		207	22.7	putative DNA-binding domain	central
			ce2007		263	30.5	putative DNA-binding domain*	N-terminal, central
			ce2201		129	14.5	putative DNA-binding domain	N-terminal
			ce2626		115	13.1	putative DNA-binding domain	N-terminal
20	PadR	4	*ce0633*		198	22.4	winged helix	N-terminal
			ce0933		181	20.4	winged helix	N-terminal
			ce2503		103	11.4	winged helix	C-terminal
			ce2815		214	24.2	winged helix	central
21	ROK	1	*ce0123*		440	46.8	winged helix	central
22	TetR	14	*ce0397*		229	25.6	homeodomain-like	N-terminal
			ce0491		172	18.3	homeodomain-like	central
			ce0665		253	28.3	homeodomain-like	central
			ce0939		223	24.5	homeodomain-like	N-terminal
			ce0985		211	23.1	homeodomain-like	central
			ce1033		250	27.8	homeodomain-like	central
			ce1663		188	21.2	homeodomain-like	N-terminal
			ce2009		288	30.9	homeodomain-like	central
			ce2287		319	35.0	homeodomain-like	central
			ce2338		247	28.0	homeodomain-like	central
			ce2388		232	25.9	homeodomain-like	N-terminal
			ce2781		222	25.3	homeodomain-like	N-terminal
			ce2788		212	23.7	homeodomain-like	N-terminal
			ce2910	*mtrR*	186	21.8	homeodomain-like	N-terminal
23	WhiB	4	*ce0283*		116	12.8	C-terminal α-helix	C-terminal
			ce0604		196	21.7	C-terminal α-helix	C-terminal
			ce0758		86	95.8	C-terminal α-helix	C-terminal
			ce0783		86	95.8	C-terminal α-helix	C-terminal
24	YbaD	1	*ce1820*		174	19.7	Zinc β-ribbon	N-terminal
25	unclassified	2	*ce0315*		123	13.7	winged helix	N-terminal
			ce1292		251	27.1	winged helix	N-terminal

* Two DNA-binding domains were identified.
