# Supplementary material for: The individual and common repertoire of DNA-binding transcriptional regulators of Corynebacterium glutamicum, Corynebacterium efficiens, Corynebacterium diphtheriae and Corynebacterium jeikeium deduced from the complete genome sequences
Source: BMC Genomics. 2005 Jun 7;6:86. doi: 10.1186/1471-2164-6-86 (PMC1180825; doi:10.1186/1471-2164-6-86)
Supplement: Additional File 3 — classification and relevant molecular data of the DNA-binding transcriptional regulators identified in C. diphtheriae NCTC 13129. [file 1471-2164-6-86-S3.pdf]

Additional file 3

DNA-binding transcriptional regulators identified in *C. diphtheriae* NCTC 13129								
			Gene		Protein		DNA-binding domain	
No.	Regulator family	Number of family members	No.	Name	Length [amino acids]	Molecular mass [kDa]	Type	Position
1	AraC	1	*dip0922*		335	36.1	homeodomain-like*	N-terminal
2	ArgR	1	*dip1172*	*argR*	163	17.5	winged helix	N-terminal
3	ArsR	3	*dip0415*		89	10.1	winged helix	central
			dip1296		238	25.6	winged helix	central
			dip1709		125	13.9	winged helix	central
4	Crp	1	*dip0303*		227	25.1	winged helix	C-terminal
5	DeoR	2	*dip1427*		258	27.3	winged helix	N-terminal
			dip1429		267	28.6	winged helix	N-terminal
6	DtxR	2	*dip0619*	*dtxR2*	204	22.6	winged helix	N-terminal
			dip1414	*dtxR*	226	25.3	winged helix	N-terminal
7	FUR	1	*dip1710*	*fur*	575	38.2	winged helix	N-terminal
8	GntR	7	*dip0011*		242	27.1	winged helix	N-terminal
			dip0226		457	49.4	winged helix	central
			dip0517		253	28.6	winged helix	N-terminal
			dip1662		227	25.6	winged helix	N-terminal
			dip2081		232	25.7	winged helix	N-terminal
			dip2241		278	30.6	winged helix	central
			dip2280		128	13.9	winged helix	central
9	HrcA	1	*dip1721*		345	37.2	winged helix	central
10	HTH_3	6	*dip0086*		99	11.2	λ repressor-like	central
			dip0183		340	38.2	λ repressor-like	N-terminal
			dip0187		80	8.6	λ repressor-like	central
			dip0369		474	53.2	λ repressor-like	N-terminal
			dip0643		193	21.8	λ repressor-like	central
			dip1456		109	11.5	λ repressor-like	central
11	IclR	1	*dip1126*		241	25.3	winged helix	N-terminal
12	LacI	2	*dip0656*		351	37.6	λ repressor-like	N-terminal
			dip1969		379	40.4	λ repressor-like	N-terminal
13	LexA	1	*dip1426*		237	25.7	winged helix	central
14	LuxR	1	*dip1889*		281	30.9	C-terminal effector domain	C-terminal
15	LysR	3	*dip0905*		298	31.3	winged helix	N-terminal
			dip1092		293	32.1	winged helix	N-terminal
			dip1421		312	33.7	winged helix	N-terminal
16	MarR	5	*dip0127*		156	18.1	winged helix	central
			dip1028		160	18.3	winged helix	central
			dip1879		221	23.7	winged helix	N-terminal
			dip2008		153	16.9	winged helix	central
			dip2296		150	17.1	winged helix	central
17	MerR	4	*dip0102*		258	29.7	putative DNA-binding domain	N-terminal
			dip1205		225	24.9	putative DNA-binding domain*	N-terminal, central
			dip1207		186	38.0	putative DNA-binding domain	central
			dip2117		132	14.7	putative DNA-binding domain	N-terminal
18	PadR	1	*dip2147*		108	12.1	winged helix	central
19	ROK	1	*dip0123*		332	35.0	winged helix	N-terminal
20	TetR	12	*dip0846*		221	24.3	homeodomain-like	N-terminal
			dip0888		124	13.8	homeodomain-like	N-terminal
			dip0910		215	23.7	homeodomain-like	N-terminal
			dip0937		215	24.6	homeodomain-like	N-terminal
			dip1117		181	19.7	homeodomain-like	N-terminal
			dip1284		190	21.8	homeodomain-like	N-terminal
			dip1358		196	21.1	homeodomain-like	N-terminal
			dip1571		181	19.5	homeodomain-like	N-terminal

			dip1788		250	27.6	homeodomain-like	N-terminal
			dip1844		214	24.1	homeodomain-like	N-terminal
			dip1942		206	22.6	homeodomain-like	N-terminal
			dip2274		208	23.6	homeodomain-like	N-terminal
21	WhiB	3	*dip0299*		115	12.9	C-terminal α-helix	C-terminal
			dip0684		99	11.2	C-terminal α-helix	C-terminal
			dip0712		86	9.4	C-terminal α-helix	C-terminal
22	YbaD	1	*dip1424*		151	17.5	Zinc β-ribbon	N-terminal
23	unclassified	3	*dip0119*		579	64.0	winged helix	C-terminal
			dip0267		343	36.9	homeodomain-like	N-terminal
			dip0494		247	27.0	winged helix	N-terminal

* Two DNA-binding domains were identified.
